# Tyrosine Phosphorylation of the E3 Ubiquitin Ligase TRIM21 Positively Regulates Interaction with IRF3 and Hence TRIM21 Activity

**DOI:** 10.1371/journal.pone.0034041

**Published:** 2012-03-30

**Authors:** Kevin B. Stacey, Eamon Breen, Caroline A. Jefferies

**Affiliations:** 1 Department Molecular and Cellular Therapeutics, Royal College Surgeons in Ireland Research Institute, Royal College of Surgeons in Ireland, Dublin, Ireland; 2 Centenary Institute and Arthur William (AW) Morrow Gastroenterology and Liver Centre, Camperdown, Australia,; Beth Israel Deaconess Medical Center, United States of America

## Abstract

Patients suffering from Systemic Lupus Erythematous (SLE) have elevated type I interferon (IFN) levels which correlate with disease activity and severity. TRIM21, an autoantigen associated with SLE, has been identified as an ubiquitin E3 ligase that targets the transcription factor IRF3 in order to turn off and limit type I IFN production following detection of viral and bacterial infection by Toll Like Receptors (TLRs). However, how the activity of TRIM21 is regulated downstream of TLRs is unknown. In this study we demonstrate that TRIM21 is tyrosine phosphorylated following TLR3 and TLR4 stimulation, suggesting that its activity is potentially regulated by tyrosine phosphorylation. Using Netphos, we have identified three key tyrosines that are strongly predicted to be phosphorylated, two of which are conserved between the human and murine forms of TRIM21, at residues 343, 388, and 393, all of which have been mutated from tyrosine to phenylalanine (Y343F, Y388F, and Y393F). We have observed that tyrosine phosphorylation of TRIM21 only occurs in the substrate binding PRY/SPRY domain, and that Y393, and to a lesser extent, Y388 are required for TRIM21 to function as a negative regulator of IFN-β promoter activity. Further studies revealed that mutating Y393 to phenylalanine inhibits the ability of TRIM21 to interact with its substrate, IRF3, thus providing a molecular explanation for the lack of activity of Y393 on the IFN-β promoter. Our data demonstrates a novel role for tyrosine phosphorylation in regulating the activity of TRIM21 downstream of TLR3 and TLR4. Given the pathogenic role of TRIM21 in systemic autoimmunity, these findings have important implications for the development of novel therapeutics.

## Introduction

Mechanisms that regulate and limit protective immune responses are critical in the prevention of excessive and pathological production of type I IFNs or pro-inflammatory cytokines. The concept that ubiquitin-mediated degradation of key targets on the pathway may be important in this context is not new, the canonical example being the regulation of NF-κB activation via the activity of the E3 ubiquitin ligases A20 and CYLD. Both A20 and CYLD limit the duration of NFκB activation via a ubiquitin editing mechanism. A20 for example has dual deubiquitinating and ubiquitinating activity and replaces K63-linked ubiquitin chains on two key signalling proteins on the NFκB pathway, IRAK1 and TRAF6, for K48 linked chains and thus targets them for proteasomal degradation [1], [2]. In a similar manner CYLD replaces K63-linked chains on the important scaffold protein on the NFκB pathway, IKK-γ, for K48-linked chains, thus targeting it for degradation and turning off the NFκB pathway [3], [4], [5].

TRIM21 (Ro52/SSA1) is a member of the TRIM (Tripartite Motif) family of single-protein E3 ligases and was originally identified as an autoantigen prevalent in Systemic Lupus Erythematosus (SLE). We have since identified TRIM21 as an E3 ligase that negatively regulates type I IFN production downstream of the pathogen recognition receptors (PRRs) TLR-3, TLR-4, TLR-7 and TLR-9, innate immune receptors specific for recognising viral and bacterial pathogens and inducing an immune response appropriate to the pathogen detected [6], [7], [8]. Both TLR3 (recognising double stranded RNA viruses) and TLR4 (gram-negative bacterial LPS) respond by inducing pro-inflammatory cytokines such as TNF-α and IL-12, in addition to the antiviral cytokines, the type I interferons (IFNs), via the activation of transcription factors such as NFκB and the interferon regulatory factors (IRFs), respectively. TRIM21 has been shown to bind and regulate the stability of the IRF family of transcription factors, including IRF3, IRF7, and IRF8, which are responsible for driving type I IFN and IL-12 expression, downstream of these receptors [6], [7], [9]. In the case of IRF3 and IRF7, TRIM21 in some studies has been shown to promote degradation of these transcription factors in order to turn off and limit the production of type I interferons [6], [7]. However, TRIM21 has also been shown to stabilise IRF3 expression levels in response to sendai virus infection at the early phase of infection [10], thus indicating that the role of TRIM21 in IRF regulation is complex. Regarding IRF8, TRIM21 has been demonstrated to interact with and ubiquitinate IRF8 leading to enhanced IL-12 production [9]. Two groups have generated TRIM21-deficient mice, and whilst both groups reported reduced ubiquitination of IRF family members and enhanced expression of inflammatory cytokines, both showed very differing phenotypes [11], [12]. In keeping with a role for TRIM21 in limiting TLR-induced inflammatory responses, Espinosa *et al* have demonstrated that lack of functional TRIM21 in mice results in enhanced levels of circulating type I IFN, IL-23, and enhanced responses to TLR7 and TLR9 ligands [12]. Interestingly, these mice develop lupus-like symptoms, including hypergamma-globulinemia, autoantibodies to DNA, proteinuria, and kidney pathology, consistent with the observations that both type I IFNs and IL-23 are elevated in lupus patients and correlate with both disease activity and pathology [12]. In contrast, when the full gene for TRIM21 was disrupted by Yoshimi *et al*, no observable phenotype in immune cells was observed, the authors suggesting that upregulation of other TRIM family members acted to compensate for the loss of TRIM21 [11]. TRIM21 is highly conserved across species with 67% homology between the human and murine forms. On balance, however it would appear that TRIM21 functions as a negative regulator of IRF3 and IRF7 mediated cytokine production, thus having important implications for systemic autoimmune diseases where type I IFNs play such an important role. Interestingly, genome wide association studies have mapped lupus susceptibility genes to the genomic region that contains TRIM21, directly linking TRIM21 with the disease [13], [14].

Phosphorylation of ubiquitin E3 ligases has previously been shown to be a key mechanism for regulating their activity. For example, Pellino3, which functions as a direct negative regulator of innate immune signalling by regulating IRAK degradation via its E3 ligase activity [15], is in turn regulated by the serine/threonine kinases IRAK1 and IRAK4, thus enhancing its E3 ubiquitin ligase activity [16]. Tyrosine phosphorylation of E3 ligases has also been implicated in the regulation of a number of different E3 ubiquitin ligases, thereby potentially affecting the ability of the E3 ligase to interact with either its substrates or its specific E2 thus regulating its activity or potentially regulating the stability of the E3 ligase itself. For example, mutation studies of c-Cbl have demonstrated a number of tyrosines critical for regulating its function. Whilst phosphorylation of C terminal tyrosine residues enhance interaction with its substrates [17], phosphorylation of Y371 within the linker region of the protein induces a conformational change in c-Cbl that enhances its activity, thus breaking a negative intramolecular interaction. Recent evidence also demonstrates that phosphorylation of this tyrosine is critical for reducing the affinity for c-Cbl for the E2 enzyme UbcH5b, resulting in increased E3 ligase activity of c-Cbl [17], [18], [19].

To date however the mechanism by which TRIM21 activity is regulated has not been investigated. Our work suggests that tyrosine phosphorylation of TRIM21 positively regulates its activity and identify tyrosine 393 in its PRY/SPRY domain as the critical tyrosine in this respect. Critically, mutation of tyrosine 393 to phenylalanine results in the loss of activity of TRIM21 and reduces its ability to interact with its substrate, IRF3. Our data therefore suggests a novel mechanism by which TRIM21 activity is regulated via promoting interaction with its substrate and provides insights into TRIM21 activity that may be manipulated in order to regulate type I IFN production in the treatment of autoimmune disorders such as SLE, in which overproduction of type I IFN and IL-23 play an important role in the pathogenesis of the disease.

## Results and Discussion

### TRIM21 is tyrosine phosphorylated in response to TLR stimulation

**Figure 1:**
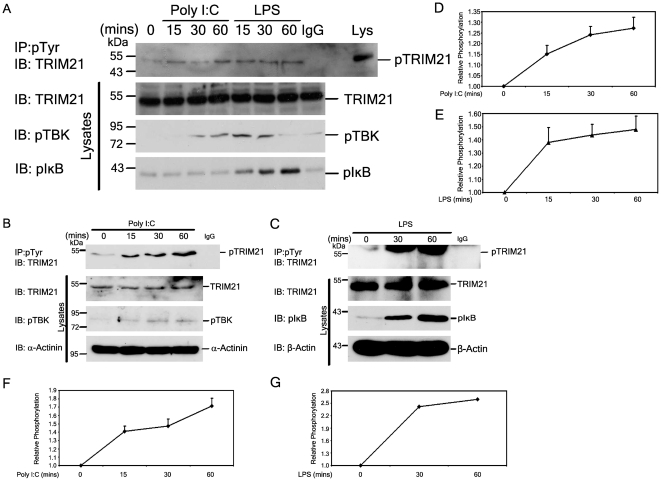
TRIM21 is tyrosine phosphorylated following TLR3 and TLR4 stimulation. (A) Immortalised BMDMs and (B, C) human PBMCs were treated with indicated TLR ligands for the indicated time course. Tyrosine phosphorylated proteins were immunoprecipitated from lysates with α-phospho-tyrosine agarose beads (Sigma). Tyrosine phosphorylation was assessed by immunoblotting for TRIM21. Figures are representative of 3 independent experiments. (D–G) Densitometric analysis was carried out on the western blots and the relative phosphorylation level of TRIM21 in comparison to phospho-TRIM21 levels in resting cells was graphed from the average of three separate experiments ± standard error of the mean (S.E.M).

**Figure 2:**
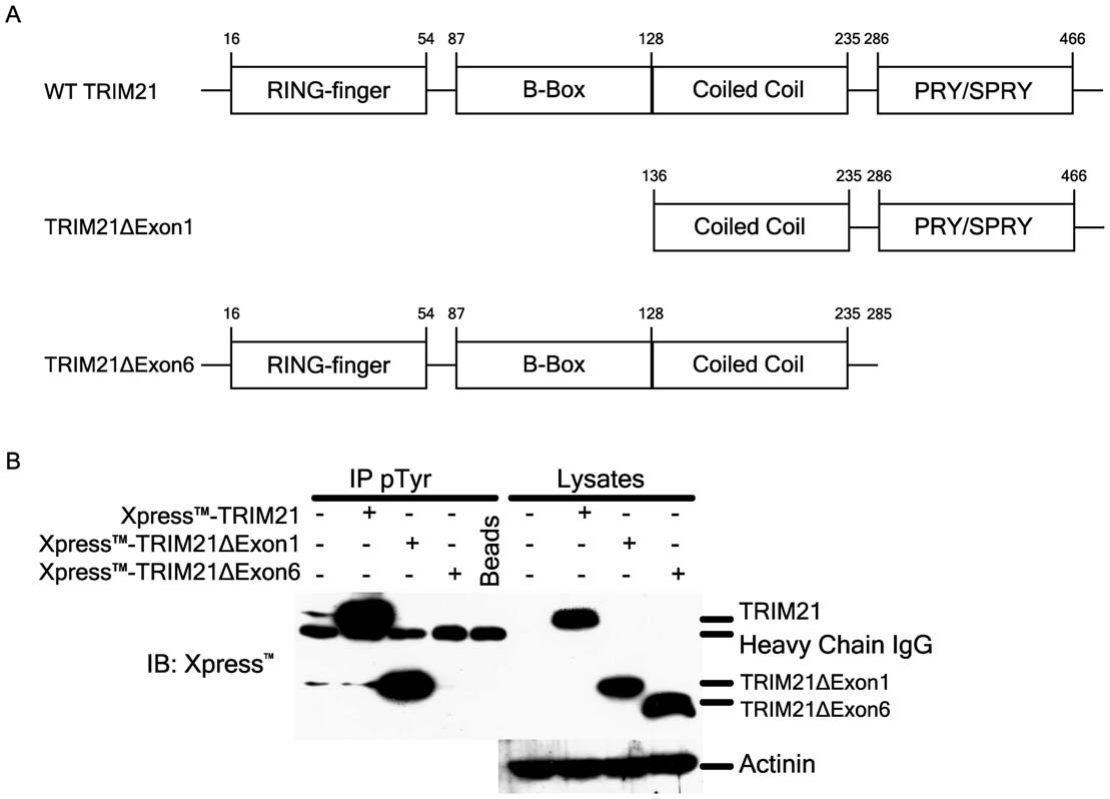
TRIM21 is tyrosine phosphorylated in the PRY/SPRY domain. (A) Schematic outline of WT TRIM21, TRIM21ΔExon1, and TRIM21ΔExon6. (B) Tyrosine phosphorylated proteins were immunoprecipitated as before from cell lysates from HEK293T cells overexpressing TRIM21 constructs and tyrosine phosphorylation of TRIM21 was assessed by immunoblotting with the anti-Xpress™ antibody (Invitrogen).

Work by our group and others has demonstrated a critical role for TRIM21 in regulating TLR–driven cytokine production [6], [9], [12]. To investigate potential means of regulating TRIM21 activity we investigated the possibility that TLR-stimulation of cells induced tyrosine phosphorylation of TRIM21. Immortalised murine Bone-Marrow Derived Macrophages (BMDMs) were stimulated with both Poly I:C (20 µg/ml), a TLR3 agonist, and LPS (1 µg/ml), a TLR4 agonist, for 0, 15, 30, and 60 mins. Immunoprecipitation of tyrosine phosphorylated proteins from cell lysates was performed and the presence of tyrosine phosphorylated endogenous TRIM21 was determined by western blotting. As can be seen in figure 1a, upper panel, both Poly I:C and LPS stimulation resulted in a rapid and sustained phosphorylation of TRIM21, with phospho-TRIM21 detectable as early as 15 mins post-stimulation. Both TLR3 and TLR4 stimulation activated the cells as expected as measured by the induction of phospho-TBK-1 and phospho-IκB, key proteins on the IRF3 and NFκB pathways, respectively (figure 1a lower panels). Figure 1d and 1e show the relevant densitometry analysis of these western blots clearly showing the increase in tyrosine phosphorylation over time following TLR3 and TLR4 stimulation respectively in murine BMDMs. Tyrosine phosphorylation levels are normalised to phospho-TRIM21 levels in resting cells. The plots are graphed from the average of three separate experiments ± standard error of the mean. Recent work carried out by James *et al* has determined TRIM21 to be an IgG receptor that binds normal serum IgG through its PRY/SPRY domain [20]. Although unlikely given the fact that Rhodes *et al* have recently shown that mouse Abs do not bind to human TRIM21 [21], isotype-specific IgG-agarose was used as a negative control in all immunoprecipitation experiments as can be seen in figure 1 and figure 2.

Having demonstrated that TRIM21 is tyrosine phosphorylated in murine macrophages we next assessed its ability to be similarly regulated in human peripheral blood mononuclear cells (PBMCs). Accordingly PBMCs isolated from healthy volunteers were stimulated with the TLR3 agonist Poly I:C for 0, 15, 30 and 60 minutes as indicated in figure 1b, and the TLR4 agonist LPS for 0, 30, and 60 minutes as indicated in figure 1c, and the phospho-status of TRIM21 determined as before. As with the murine system, very low levels of tyrosine phosphorylated TRIM21 is present in resting cells and stimulation with both TLR3 and TLR4 agonists induced a rapid and sustained phosphorylation of the E3 ligase, thus confirming the potential importance of tyrosine phosphorylation of human TRIM21 in regulating its activity. Figures 1f and 1g show the relevant densitometry analysis of the western blot of figures 1b and 1c clearly showing the increase in tyrosine phosphorylation over time following TLR3 and TLR4 stimulation in primary human PBMCs. Tyrosine phosphorylation levels are normalised to phospho-TRIM21 levels in resting cells. The plots are graphed from the average of three separate experiments ± standard error of the mean. A role for tyrosine kinases in LPS signalling has been known since the first observation in 1991 that LPS treatment of murine macrophages induced the expression of protein tyrosine kinases Hck and Lyn [22]. In addition, TLR2 and 3 have been reported to be inducibly phosphorylated on tyrosine residues on ligand binding and one report has identified a tyrosine in MyD88 that is inducibly phosphorylated [23]. In each case the phosphorylated tyrosine reportedly binds the p85 adaptor subunit of phosphatidylinositol 3-kinase (PI3-K) and through it positively regulates NFκB activity. Bruton's tyrosine kinase (Btk) has been shown to be activated downstream of TLR4 and TLR8 and 9, resulting in enhanced activation of NFκB and hence enhanced pro-inflammatory cytokine production [24], [25], [26]. In addition Btk has been shown to phosphorylate the adaptor molecule MyD88 Adaptor-like (Mal) post TLR2 and TLR4 activation, again regulating NFκB activation [27]. As yet, the nature of the kinase responsible for phosphorylating TRIM21 is unknown, although it is tempting to speculate that Btk may be involved, acting post-TLR stimulation to phosphorylate and hence regulate this critical E3 ubiquitin ligase and promote its negative effects on TLR-induced IFN production

### TRIM21 is tyrosine phosphorylated on its PRY/SPRY domain – a domain critical for interaction with substrates

Like other TRIM family members, TRIM21 is a single protein E3 ligase with an N terminal RING domain (for interaction with its cognate E2 enzyme), a central coiled-coil domain, and a C terminal substrate interaction domain, in the case of TRIM21 a PRY/SPRY domain. To determine which domain of TRIM21 was tyrosine phosphorylated we next performed overexpression studies in HEK-293T cells, employing TRIM21 constructs missing the RING domain (Δexon1) or the C terminal PRY/SPRY domain (Δexon6) (figure 2a). As with endogenous TRIM21, tyrosine phosphorylated proteins were enriched from cell lysates and the presence of phosphorylated TRIM21 detected by western blotting. As shown in figure 2b, full-length TRIM21 was tyrosine phosphorylated following overexpression as expected. However, whilst the mutant lacking the N-terminal RING domain was tyrosine phosphorylated, the PRY/SPRY deletion mutant demonstrated a complete inability to become phosphorylated, indicating that tyrosine phosphorylation of TRIM21 occurs in this region of the protein. The PRY/SPRY domain of TRIM21 is well known to be essential for binding of substrates, such as IRF3, IRF7, and IRF8 [7], [9], [28]. We have demonstrated here that TRIM21 is tyrosine phosphorylated only in this domain indicating a possible role for the tyrosine phosphorylation of TRIM21 regulating its ability to bind to IRF3, thus controlling further downstream affects of TRIM21 activity.

**Figure 3:**
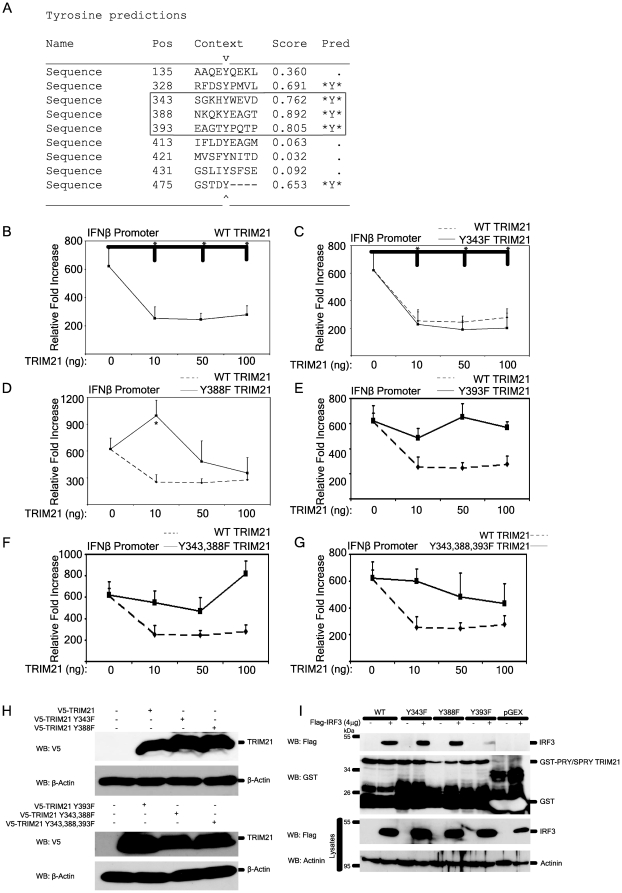
Tyrosine 393 regulates IRF3 interaction and hence the activity of TRIM21. (A), Netphos data predicting potential tyrosine phosphorylation sites in TRIM21. (B–G) TRIF-driven IFN-β reporter gene analysis was measured in HEK293T cells transfected with indicated TRIM21 plasmids. Kruskal-Wallis ANOVA analysis assessed relative fold increase of IFN-β promoter activity. * *P*<0.05 was considered significant and is compared to TRIF driven IFN-β promoter activity alone. (H) Cell lysates prepared from HEK293T cells overexpressing WT TRIM21 and the indicated mutants were assessed for expression of TRIM21 by western blotting with the anti-V5 antibody (Invitrogen). (I) Cell lysates prepared from HEK293T cells overexpressing FLAG-IRF3 were incubated with recombinant GST-PRY/SPRY TRIM21 pre-coupled to GSH-sepharose beads as indicated and the interaction between TRIM21and IRF3 assessed by immunoblotting. Figures are representative of three separate experiments.

***Y393 is important for TRIM21 function*** **– Using NetPhos** (http://www.cbs.dtu.dk/services/NetPhos/), tyrosine 343, 388 and 393 were strongly predicted to be phosphorylated (as shown by the NetPhos score, figure 3a) and were subsequently mutated to phenylalanine in order to assess their role in regulating TRIM21 function. Y343 and Y388 are conserved between human and murine TRIM21 and are also predicted to be phosphorylated. Interestingly, Y393 in human TRIM21 corresponds to S396 in murine TRIM21 and this serine residue is also predicted to be phosphorylated in murine TRIM21 (data not shown). As TRIM21 has previously been demonstrated to be a potent inhibitor of TLR-driven IFN-β production [6], we employed an IFN-β promoter reporter gene assay driven by an upstream activator of the pathway, the adaptor protein TRIF, and assessed the effects of each of the tyrosine mutants in this system. As expected from previous reports, wild type full length TRIM21 dose dependently inhibited IFN-β promoter activity, in keeping with its role as a negative regulator of this response (figure 3b). Similarly transfection of cells with increasing amounts of Y343F resulted in a similar inhibitory response, indicating that Y343 is not essential for TRIM21 activity (figure 3c). In contrast we observed a lack of inhibitory activity when Y388F and to a greater extent Y393F mutants were assessed (figure 3d and 3e, respectively). Further analysis to assess the effects of double (Y343,388F) and triple (Y343,388,393F) mutants of TRIM21 was also conducted, with both the double and triple mutants inhibiting the response in a similar manner observed in the Y393F mutant as seen in figure 3f and 3g respectively. To ensure all plasmids were expressing to a similar level, expression analysis of the mutants was carried out. Figure 3h illustrates that Y393F expresses to a higher level than the other TRIM21 plasmids. However the increased expression of Y393F does not lead to an increase in inhibition of IFN-β promoter activity, emphasising that the lack of inhibition in this regard is as a result of the tyrosine to phenylalanine mutation. All other TRIM21 plasmids express to a similar level within the cell, again suggesting that changes observed with these mutants in their ability to inhibit TRIF-driven IFN-β promoter activity is indeed as a result of the tyrosine to phenylalanine mutations and not due to differences in protein expression. These results suggest that tyrosine phosphorylation of Y393 is required for the activity of TRIM21 in this context to be maintained. Previous reports of tyrosine phosphorylation of E3 ligases such as c-Cbl have shown that this modification can either alter intramolecular interactions within the protein or enhance interaction with its substrate [17], [18]. Tyrosine phosphorylation may also alter subcellular localisation of a protein or its stability [29]. Expression analysis of our mutants indicated that stability of TRIM21 was not affected (figure 3h), and given the fact that tyrosine phosphorylation regulated the activity of the E3 ligase and occurred in the PRY/SPRY domain, we hypothesised that phosphorylation at Y393 and possibly Y388 may alter substrate interaction.

### Y393 is essential for facilitating the interaction between TRIM21 and its substrate IRF3

Importantly, based on the published crystal structure of TRIM21 [20], Y388 and Y393 are associated with a known substrate binding pocket and thus phosphorylation at these sites may regulate interactions between TRIM21 and its known substrates such as IRF3. To test this hypothesis, recombinant PRY/SPRY domain TRIM21 (wild type and tyrosine mutants) was constructed for use in pull-down experiments. This would ensure that any observed effect of mutating the individual tyrosines in TRIM21 on IRF3 binding was indeed a result of altered affinity between the two proteins due to the mutation and not an artefact (however unlikely) based on non-specific binding of TRIM21 to antibody. Therefore recombinant PRY/SPRY TRIM21 proteins (wild type, PRY/SPRY-Y343F, PRY/SPRY-Y388F and PRY/SPRY-Y393F) were incubated individually with cell lysates from HEK293T cells overexpressing FLAG-tagged IRF3 and the ability of the recombinant protein to interact with IRF3 assessed by western blotting. As shown in figure 3i, lane 2, wild type TRIM21 interacts with IRF3 as expected. In keeping with our reporter gene analysis, PRY/SPRY-Y343F also interacted with IRF3 (lane 4), confirming a lack of any functional role for this tyrosine in regulating the ability of TRIM21 to interact with IRF3 and thus inhibit its activity. PRY/SPRY-Y388F also interacted strongly with IRF3, indicating that the reduced activity of Y388F, at lower doses of plasmid DNA, on IFN-β promoter activity was not related to its ability to interact with its substrate on this pathway IRF3 (lane 6). Perhaps further studies involving dose-response experiments may elucidate a role for Y388 in the interaction of TRIM21 and IRF3. However, PRY/SPRY-Y393F had a greatly reduced ability to interact with IRF3 (lane 8), suggesting that the lack of inhibitory effect of this mutant on the IFN-β promoter was as a direct result of the clear reduction in interaction occurring between the mutant and the substrate IRF3. From analysis of the crystal structure of the PRY/SPRY domain of TRIM21 elucidated by James *et al*, Y388 resides within an IgG binding pocket and Y393 resides just outside this pocket [20]. Our results would indicate that the interaction of TRIM21 and IRF3 is reliant on Y393, and that the role that Y388 plays in the regulation of TRIM21 activity is yet to be determined. Our results would also indicate that the mutation of Y388 does not affect the ability of TRIM21 to interact with IRF3, suggesting that the binding site of IRF3 is not at Y388, but perhaps at Y393 which resides at a much more accessible location in the PRY/SPRY domain of TRIM21.

Thus our studies reveal a novel mechanism for regulating the activity of the E3 ligase TRIM21 via tyrosine phosphorylation of tyrosine 393 thus promoting its interaction with its substrate IRF3. In this respect, tyrosine phosphorylation of TRIM21 at this site functions to turn on and off its activity. Recently a number of groups have demonstrated the importance of TRIM21 in regulating IFN and proinflammatory cytokine production downstream of anti-viral detection systems, via its ability to target the IRF family of transcription factors [6], [7], [9]. Importantly, genetic deletion of TRIM21 in mice resulted in the development of a systemic autoimmune condition as a result of overproduction of type I IFNs and the Th17 cell promoting cytokine, IL-23 [12]. Recently we have shown that TRIM21 acts as a negative regulator of IL-23 downstream of TLR3 stimulation [30] and Al-Salleeh *et al* have previously demonstrated a similar role for TRIM21 downstream of TLR7 and TLR9 activation [31]. Thus any mechanism resulting in reduced activity of TRIM21 (whether by presence of inactivating mutations in *TRIM21*, or via the ability of autoantibodies against TRIM21 to inhibit E2 interactions [32]) would have the effect of reducing the effectiveness of TRIM21 as a guardian against overproduction of type I IFNs and potentially IL-23, thus contributing directly to the pathology of SLE. In this respect, TRIM21 is an important target in the treatment of SLE. Our novel findings regarding the regulation of TRIM21 activity by tyrosine phosphorylation may have important implications with regards to its possible manipulation in order to treat systemic autoimmune conditions such as SLE. As with other E3 ligases that are regulated by phosphorylation such as c-Cbl and the Pellino family, knowledge regarding the kinases involved and the target sites will have a direct impact on rational drug design in order to manipulate their activity.

## Materials and Methods

### Cell Culture

The HEK293T cell line and the J2 immortalised Bone-Marrow derived macrophages (BMDMs) from wild type mice, a kind gift from Dr. Egil Lien and Prof. Douglas Golenbock (UMASS Medical School, Worcester, MA), were cultured in Dulbecco's modified Eagle's medium supplemented with 10% (v/v) fetal calf serum and 1% penicillin-streptomycin. Primary human peripheral mononuclear cells (PBMCs) were isolated from whole blood from healthy donors, under ethical approval from Royal College of Surgeons in Ireland research ethics committee REC269, using a Ficoll gradient and cultured in RPMI-1640 media supplemented with 10% (v/v) fetal calf serum and 1% penicillin-streptomycin. Informed consent from all participants involved in this study was obtained in a written manner. Participants involved in this study were only recruited from, and experimentation conducted at, Royal College of Surgeons in Ireland.

### Plasmids and reagents

Xpress-tagged TRIM21, Xpress-tagged TRIM21ΔExon1, Xpress-tagged TRIM21ΔExon6, and pGEX-PRY/SPRY TRIM21 were gifts from Dr. David Rhodes (Cambridge Institute for Medical Research, Cambridge, U.K.), and the Flag-tagged pCMV-IRF3 and the IFN-β promoter construct were gifts from Dr. Kate Fitzgerald (University of Massachusetts Medical School, Worcester, MA). TRIM21 Y393F, TRIM21 Y343,388F, and TRIM21 Y343,388,393F were generated by Genscript USA Inc. (Piscataway, NJ 08854). Polyclonal Ab against TRIM21 was made by Sigma Genosys. Primary antibodies used were anti-Xpress, anti-V5 (Invitrogen Life Technologies), anti-phospho-IκB (Cell Signalling), anti-phospho-TBK and anti-alpha-actinin (Santa Cruz Biotechnologies), anti-Flag (Sigma), anti-GST (GE Healthcare), and anti-β-actin (Abcam). Anti-phosphotyrosine agarose beads were obtained from Sigma.

### Site-directed mutagenesis

V5-TRIM21 was generated from cDNA and inserted into pcDNA3.1 using the Gateway™ vector system (Invitrogen Life Technologies). The V5-TRIM21 and the pGEX-PRY/SPRY TRIM21 tyrosine to phenylalanine mutants were generated from the V5-TRIM21 plasmid and pGEX-PRY/SPRY TRIM21 plasmid using the QuikChange Lightning site-directed mutagenesis kit (Stratagene). The primers used for the mutagenesis are as follows: Y343F, cactctggaaaacatttctgggaggtagatgtg (forward) and cacatctacctcccagaaatgttttccagagtg (reverse); Y388F, gtggaacaaacaaaaattcgaggctggcacctaccc (forward) and gggtaggtgccagcctcgaatttttgtttgttccac (reverse); Y393F, gggattttcctggacttcgaggctggcatggtc (forward) and gaccatgccagcctcgaagtccaggaaaatccc (reverse).

### Immunoprecipitation and Western Blot Analysis

Immunoblots were performed as previously described [25]. Cells were lysed on ice in 1× Tris-HCl buffer (50 mM Tris-HCl, pH 7.4, 1% Nonidet P40, 0.25% (w/v) sodium deoxycholate, 150 mM NaCl, 1 mM EDTA, 1 mM phenylmethylsulfonyl fluoride, 1 mM Na_3_VO_4_, 1 µg/ml pepstatin) followed by immunoprecipitation with anti-phosphotyrosine-conjugated agarose beads (Sigma). For recombinant pulldowns, lysates were incubated with 500 ng of GST-PRY/SPRY TRIM21 bound to GSH-sepharose beads (Qiagen). Immunoprecipitates were analysed by Western blot. Each blot is representative of three independent experiments.

### Reporter Gene Assays

HEK293T cells were seeded (10^5^ cells/ml) onto 96-well plates 24 hrs pre-transfection with 50 ng of the indicated reporter constructs and increasing amounts of the relevant TRIM21 constructs (10, 50, and 100 ng) as indicated. HEK293T cells were also co-transfected with TRIF (50 ng) in order to drive the system. Luciferase activity was standardised to *Renilla* luciferase plasmid activity to normalise for transfection efficiency.

### Statistical Analysis

A Kruskal-Wallis one-way analysis of variance was carried out on non-parametric data using PASW Statistics version 18 from SPSS (IBM Corporation, New York, USA). *P*<0.05 was considered to be of significance.
